# 

**Published:** 1877-06-20

**Authors:** 


					﻿VOL. VII.	. JUNE 20, 1877	No. 6.
THE
SOUTHERN MEDICAL RECORD.
A MONTHLY JOURNAL OF PRACTICAL MEDICINE.
QUJCQU1D rii^CiriES ESTO BliEVIS.”
W
H
w
Ph
O
f—i
H
W
—
p
o
c
Ph
o
Hs
02
)—<
E
f-H
Ph
q
GQ
>4
o
w
w
&
M
W
£
w
rZ)
<;
w
d
Oh
CO
M
o
w
H
Q
O
t>
i—i
te«
d
HH
Q
>
h3
l-H
o
00'
>
•n
>
o
h
hH
Q
d
i—i
i-3
M
t>
HH
CO
CO
o
d
HH
a
l-H
W
U
JAS. P. HARRISON & CO., Printers.
ATLANTA, GEORGIA.
TERMS: $2.00 per Annum in Advance—Club Rates, 5 Copies for $8.00.
				

